# Sex-differences in the effect of obstructive sleep apnea on patients hospitalized with pulmonary embolism and on in-hospital mortality

**DOI:** 10.1038/s41598-021-97923-y

**Published:** 2021-09-15

**Authors:** Javier de-Miguel-Diez, Marta Lopez-Herranz, Valentín Hernandez-Barrera, David Jimenez, Manuel Monreal, Rodrigo Jiménez-García, Ana López-de-Andrés

**Affiliations:** 1 Pneumology Department, Hospital General Universitario Gregorio Marañón, Universidad Complutense de Madrid, CIBER de Enfermedades Respiratorias (CIBERES), Madrid, Spain; 2 Faculty of Nursing, Physiotherapy and Podology, Universidad Complutense de Madrid, 28040 Madrid, Spain; 3 Preventive Medicine and Public Health Teaching and Research Unit, Department of Medical Specialties and Public Health, Universidad Rey Juan Carlos, Alcorcón, Madrid, Spain; 4 Respiratory Department, Ramón y Cajal Hospital and Instituto Ramón y Cajal de Investigación Sanitaria (IRYCIS), Madrid, Spain; 5 Medicine Department, Universidad de Alcalá, CIBER de Enfermedades Respiratorias (CIBERES), Madrid, Spain; 6 Department of Internal Medicine, Hospital Universitari Germans Trias i Pujol. CIBER de Enfermedades Respiratorias (CIBERES), Badalona, Barcelona, Spain; 7 Department of Public Health & Maternal and Child Health, Faculty of Medicine, Universidad Complutense de Madrid, Madrid, Spain

**Keywords:** Epidemiology, Respiratory tract diseases

## Abstract

We determined sex differences in the prevalence of obstructive sleep apnea (OSA) among patients hospitalized with pulmonary embolism (PE) in Spain (2016–2018). We also compared outcomes according to the presence of OSA, and identified variables associated with in-hospital-mortality (IHM) after PE using the Spanish National Hospital Discharge Database. We identified 46,794 hospital admissions for PE; of these, 5.47% had OSA. OSA was more prevalent among men than women (7.57% vs. 3.65%, p < 0.001), as in the general population. Propensity score matching did not reveal differences in concomitant conditions or procedures between patients with and without OSA, except for the use of non-invasive ventilation, which was more frequent in patients with OSA. IHM was similar in patients with and without OSA (3.58% vs. 4.31% for men and 4.39% vs. 4.93% for women; p > 0.05). Older age, cancer, atrial fibrillation, non-septic shock, and need for mechanical ventilation increased IHM in men and women with OSA hospitalized with PE. The logistic regression model showed no sex differences in IHM among patients with OSA.

## Introduction

Pulmonary embolism (PE) generates a substantial burden for public health services because of its high morbidity and mortality. It is the most life-threatening form of venous thromboembolism and the third most frequent cause of vascular death worldwide^1,2^. The main risk factors for PE are age, active cancer, congenital or acquired thrombophilia, hormone replacement and oral contraceptive therapy, previous PE, and obesity. However, up to 30% of cases remain unexplained^3^.

Obstructive sleep apnea (OSA), which is characterized by recurrent upper airway obstruction during sleep with intermittent hypoxia and sleep fragmentation^4^, is an independent risk factor for cardiovascular morbidity and death^5^. Various studies point to a possible association between OSA and PE^6–9^. Some of the mechanisms via which OSA can contribute to the development of venous thromboembolism include sympathetic hyperactivity, oxidative stress, systemic inflammation, hemodynamic abnormalities, and vascular endothelial dysfunction resulting in hypercoagulability, vascular damage, and venous stasis^10–12^. Despite increased knowledge of the relationship between OSA and PE, no studies to date have explored sex differences in the prevalence and mortality of OSA in patients hospitalized with PE.

OSA seems to increase the risk of PE and or recurrences^13^, yet data on the association between OSA and prognosis of PE patients are contradictory. Thus, some authors found no association between OSA and 30-day mortality in PE patients^14^, whereas others have suggested that OSA is a predictor of PE-related death^15^.

The objectives of this study were as follows: (a) to determine sex differences in the prevalence of OSA among patients hospitalized with PE; (b) to determine and compare clinical characteristics, use of therapeutic procedures, and in hospital mortality (IHM) among men and women with PE according to the presence of OSA; and (c) to identify which variables are independently associated with IHM after PE in men and women with OSA.

## Results

During the study period (2016–2018), 46,794 patients aged ≥ 18 years were discharged from Spanish hospitals with a primary diagnosis of PE. Of these, 2561 (5.47%) had a code for OSA. The prevalence of OSA was over twice as high in men as in women (7.57% vs. 3.65%; p < 0.001) (Table 1).

**Table 1:** Distribution and prevalence by sex, age, clinical characteristics, and hospital outcomes of patients hospitalized with pulmonary embolism (PE) according to the presence of obstructive sleep apnea.

	Distribution			OSA prevalence	
	OSA	No OSA	p-value^a^	%	p-value^b^
Total	2561 (5.47)	44,233 (94.53)		5.47	–
Men, n (%)	1649 (64.39)	20,133 (45.52)	< 0.001	7.57	< 0.001
Women, n (%)	912 (35.61)	24,100 (54.48)		3.65	
**Age, mean (SD)**	68.38 (12.21)	70.77 (15.85)	< 0.001	NA	–
18–54 years	372 (14.53)	7180 (16.23)	< 0.001	4.93	< 0.001
55–64 years	480 (18.74)	5892 (13.32)		7.53	
65–74 years	836 (32.64)	9578 (21.65)		8.03	
75–84 years	708 (27.65)	12,778 (28.89)		5.25	
85 years or over	165 (6.44)	8805 (19.91)		1.84	
CCI, mean (SD)	1.10 (0.97)	0.83 (0.73)	< 0.001	NA	–
CCI = 0, n (%)	884 (34.52)	19,405 (43.87)	< 0.001	4.36	< 0.001
CCI 1–2, n (%)	899 (35.1)	15,915 (35.98)		5.35	
CCI > 2, n (%)	778 (30.38)	8913 (20.15)		8.03	
LOHS days, median (IQR)	8 (6)	7 (6)	0.015	NA	–
IHM, n (%)	99 (3.87)	2897 (6.55)	< 0.001	NA	–

*OSA* Obstructive sleep apnea, *CCI* Charlson comorbidity index, *LOHS* Length of hospital stay, *IHM* In-hospital mortality, *SD* Standard deviation, *IQR* Interquartile range, *NA* Not applicable.

^a^P value for comparison of OSA with non-OSA patients.

^b^P value for association of the prevalence of OSA according to sex, age groups, and CCI categories.

Patients with OSA were younger than those without OSA (68.38 vs. 70.77 years; p < 0.001). The prevalence of OSA among patients with PE increased with age, reaching its highest value in patients aged 65–74 years (8.03%) and decreasing thereafter, with the lowest prevalence being found in those aged ≥ 85 years (1.84%).

The mean Charlson comorbidity index (CCI) value was 1.10 in patients with OSA and 0.83 in those without OSA (p < 0.001). The prevalence of OSA rose in line with the number of comorbidities included in the CCI from 4.36% among patients with CCI 0–8.03% in those with CCI > 2 (p < 0.001).

Regarding hospital outcomes, patients with OSA had a significantly higher median length of hospital stay (LOHS) (8 days vs. 7 days; p = 1.015) and lower crude IHM (3.87% vs. 6.55; p < 0.001).

Table 2 shows the results of our comparison of the distribution of comorbidities, therapeutic procedures, and hospital outcomes in men hospitalized for PE with and without OSA.

**Table 2:** Prevalence of specific comorbid conditions, therapeutic procedures, and hospital outcomes in men hospitalized with pulmonary embolism (PE) according to the presence of obstructive sleep apnea in Spain from 2016 to 2018 before and after propensity score matching.

	Before propensity score matching			After propensity score matching		
	OSA	No OSA	p-value	OSA	No OSA	p-value
Age, mean (SD)	66.72 (12.07)	67.56 (15.26)	0.028	66.72 (12.07)	67.02 (13.14)	0.487
CCI, mean (SD)	1.15 (1.01)	0.88 (0.87)	< 0.001	1.15 (1.01)	1.11 (0.97)	0.314
AMI, n (%)	67 (4.06)	644 (3.2)	0.058	67 (4.06)	63 (3.82)	0.720
Heart failure, n (%)	213 (12.92)	1756 (8.72)	< 0.001	213 (12.92)	203 (12.31)	0.600
PVD, n (%)	103 (6.25)	902 (4.48)	0.001	103 (6.25)	80 (4.85)	0.080
CVD, n (%)	65 (3.94)	619 (3.07)	0.052	65 (3.94)	58 (3.52)	0.520
Dementia, n (%)	45 (2.73)	591 (2.94)	0.632	45 (2.73)	41 (2.49)	0.662
Rheumatic disease, n (%)	29 (1.76)	271 (1.35)	0.167	29 (1.76)	22 (1.33)	0.323
Liver disease, n (%)	121 (7.34)	1120 (5.56)	0.003	121 (7.34)	109 (6.61)	0.412
Diabetes, n (%)	382 (23.17)	2888 (14.34)	< 0.001	382 (23.17)	388 (23.53)	0.805
COPD, n (%)	490 (29.71)	3374 (16.76)	< 0.001	490 (29.71)	530 (32.14)	0.132
Renal disease, n (%)	180 (10.92)	1550 (7.7)	< 0.001	180 (10.92)	166 (10.07)	0.426
Cancer, n (%)	226 (13.71)	3995 (19.84)	< 0.001	226 (13.71)	207 (12.55)	0.327
Atrial fibrillation, n (%)	185 (11.22)	1585 (7.87)	< 0.001	185 (11.22)	173 (10.49)	0.502
Valvular heart disease, n (%)	93 (5.64)	1005 (4.99)	0.248	93 (5.64)	89 (5.4)	0.760
Obesity, n (%)	584 (35.42)	1568 (7.79)	< 0.001	584 (35.42)	585 (35.48)	0.971
Coagulopathy, n (%)	33 (2)	429 (2.13)	0.725	33 (2)	18 (1.09)	0.034
Pulmonary hypertension, n (%)	164 (9.95)	1482 (7.36)	< 0.001	164 (9.95)	150 (9.1)	0.406
Supplemental oxygen, n (%)	113 (6.85)	453 (2.25)	< 0.001	113 (6.85)	98 (5.94)	0.286
Inferior vena cava filter, n (%)	11 (0.67)	207 (1.03)	0.157	11 (0.67)	16 (0.97)	0.334
Undergone surgery, n (%)	25 (1.52)	376 (1.87)	0.307	25 (1.52)	35 (2.12)	0.193
Thrombolytic therapy, n (%)	92 (5.58)	1258 (6.25)	0.278	92 (5.58)	88 (5.34)	0.759
NIV, n (%)	85 (5.15)	210 (1.04)	< 0.001	85 (5.15)	30 (1.82)	< 0.001
IV, n (%)	16 (0.97)	284 (1.41)	0.140	16 (0.97)	21 (1.27)	0.408
Non septic shock, n (%)	9 (0.55)	188 (0.93)	0.110	9 (0.55)	12 (0.73)	0.511
Vasopressors, n (%)	3 (0.18)	41 (0.2)	0.850	3 (0.18)	5 (0.3)	0.479
LOHS, median (IQR)	7 (6)	7 (6)	0.121	7 (6)	7 (6)	0.826
IHM, n (%)	59 (3.58)	1301 (6.46)	< 0.001	59 (3.58)	71 (4.31)	0.283

*OSA* Obstructive sleep apnea, *CCI* Charlson comorbidity index, *AMI* acute myocardial infarction, *PVD* peripheral vascular disease, *CVD* cerebrovascular disease, *COPD* chronic obstructive pulmonary disease, *NIV* non-invasive ventilation, *IV* invasive ventilation, *LOHS* length of hospital stay, *IHM* in-hospital mortality, *SD* standard deviation, *IQR* interquartile range.

p value for comparison of men and women with OSA.

In men with OSA admitted with PE, we recorded a remarkably high prevalence of obesity (35.42%), chronic obstructive pulmonary disease (COPD, 29.71%), diabetes (23.17%), heart failure (12.29%), and renal disease (10.92%). These values were significantly higher than in men without OSA. Pulmonary hypertension and dependence on supplemental oxygen were also more frequent among patients with OSA.

Cancer was recorded in 13.71% of men with OSA compared with 19.84% of those who did not have OSA (p < 0.001).

No differences were found in therapeutic procedures and surgery, except for non-invasive ventilation (NIV), which was more common among patients with OSA than among those without OSA (5.15% vs. 1.04% p < 0.001).

The crude IHM was significantly lower among men with OSA (3.58%) than among men without OSA (6.46%).

Propensity score matching (PSM) revealed that none of the differences in any of the conditions or procedures analyzed remained significant. Furthermore, significance was lost in the association between OSA and IHM (3.58% vs. 4.31%, p = 0.283).

The comparison between women with and without OSA regarding their comorbid conditions, procedures, and hospital outcomes before and after PSM is shown in Table 3.

**Table 3:** Prevalence of specific comorbid conditions. Therapeutic procedures, and hospital outcomes in women hospitalized with pulmonary embolism (PE) according to the presence of obstructive sleep apnea in Spain from 2016 to 2018 before and after propensity score matching.

	Before propensity score matching			After propensity score matching		
	OSA	No OSA	p-value	OSA	No OSA	p-value
Age, mean (SD)	71.38 (11.89)	73.45 (15.84)	< 0.001	71.38 (11.89)	71.84 (13.32)	0.434
CCI, mean (SD)	1.00 (0.88)	0.80 (0.79)	0.008	1.00 (0.88)	0.99 (0.85)	0.830
AMI, n (%)	17 (1.86)	294 (1.22)	0.085	17 (1.86)	18 (1.97)	0.864
Heart failure, n (%)	176 (19.3)	3155 (13.09)	< 0.001	176 (19.3)	167 (18.31)	0.590
PVD, n (%)	21 (2.3)	453 (1.88)	0.358	21 (2.3)	13 (1.43)	0.166
CVD, n (%)	28 (3.07)	848 (3.52)	0.470	28 (3.07)	26 (2.85)	0.782
Dementia, n (%)	25 (2.74)	1813 (7.52)	< 0.001	25 (2.74)	23 (2.52)	0.770
Rheumatic disease, n (%)	21 (2.3)	712 (2.95)	0.252	21 (2.3)	19 (2.08)	0.749
Liver disease, n (%)	48 (5.26)	907 (3.76)	0.020	48 (5.26)	46 (5.04)	0.832
Diabetes, n (%)	260 (28.51)	3686 (15.29)	< 0.001	260 (28.51)	265 (29.06)	0.796
COPD, n (%)	204 (22.37)	2628 (10.9)	< 0.001	204 (22.37)	198 (21.71)	0.735
Renal disease, n (%)	100 (10.96)	1867 (7.75)	< 0.001	100 (10.96)	93 (10.2)	0.594
Cancer, n (%)	67 (7.35)	3438 (14.27)	< 0.001	67 (7.35)	70 (7.68)	0.790
Atrial fibrillation, n (%)	76 (8.33)	2180 (9.05)	0.461	76 (8.33)	64 (7.02)	0.291
Valvular heart disease, n (%)	66 (7.24)	1659 (6.88)	0.680	66 (7.24)	65 (7.13)	0.928
Obesity, n (%)	468 (51.32)	3286 (13.63)	< 0.001	468 (51.32)	485 (53.18)	0.426
Coagulopathy, n (%)	15 (1.64)	414 (1.72)	0.867	15 (1.64)	10 (1.1)	0.314
Pulmonary hypertension, n (%)	140 (15.35)	2320 (9.63)	< 0.001	140 (15.35)	129 (14.14)	0.468
Supplemental oxygen, n (%)	81 (8.88)	473 (1.96)	< 0.001	81 (8.88)	67 (7.35)	0.230
Inferior vena cava filter, n (%)	10 (1.1)	194 (0.8)	0.337	10 (1.1)	9 (0.99)	0.818
Undergone surgery, n (%)	10 (1.1)	372 (1.54)	0.280	10 (1.1)	13 (1.43)	0.529
Thrombolytic therapy, n (%)	58 (6.36)	1358 (5.63)	0.353	58 (6.36)	42 (4.61)	0.100
NIV, n (%)	67 (7.35)	222 (0.92)	< 0.001	67 (7.35)	18 (1.97)	< 0.001
IV, n (%)	10 (1.1)	270 (1.12)	0.946	10 (1.1)	12 (1.32)	0.668
Non-septic shock, n (%)	10 (1.1)	248 (1.03)	0.843	10 (1.1)	10 (1.1)	0.999
Vasopressors, n (%)	2 (0.22)	52 (0.22)	0.982	2 (0.22)	1 (0.11)	0.563
LOHS, median (IQR)	8 (6)	8 (6)	0.151	8 (6)	8 (6)	0.206
IHM, n (%)	40 (4.39)	1596 (6.62)	0.007	40 (4.39)	45 (4.93)	0.579

*OSA* Obstructive sleep apnea, *CCI* Charlson comorbidity index, *AMI* acute myocardial infarction. *PVD* peripheral vascular disease, *CVD* cerebrovascular disease, *COPD* chronic obstructive pulmonary disease, *NIV* non-invasive ventilation, *IV* invasive ventilation, *LOHS* length of hospital stay, *IHM* in-hospital mortality, *SD* standard deviation, *IQR* interquartile range.

p value for comparison of men and women with OSA.

Women with concomitant OSA were younger and had a significantly higher mean CCI than women who did not have OSA (both p < 0.001). We also found that women with OSA had a very high prevalence of obesity (51.32%), diabetes (28.51%), COPD (22.37%), heart failure (19.3%), pulmonary hypertension (15.35%), renal disease (10.96%), and dependence on supplemental oxygen (8.88%). Values for these conditions were significantly lower among women who did not have OSA (Table 3). However, dementia (7.52% vs. 2.74%; p < 0.001) and cancer (14.27% vs. 7.35%; p < 0.001) were more frequent among women without OSA.

NIV was 8 times more frequent among women with OSA (7.35% vs. 0.92%; p < 0.001). No differences were found among women for the remaining therapeutic procedures (Table 3).

Fewer women with OSA died in hospital than women without OSA (4.39% vs. 6.62%; p < 0.001). However, as with men, after PSM, the differences in IHM became non-significant (4.39% vs. 4.93%, p = 0.579).

The data shown in Table 4 are the results of comparing men and women with PE and OSA before and after PSM. These help to assess sex-differences in the co-occurrence of PE and OSA.

**Table 4:** Prevalence of specific comorbid conditions, therapeutic procedures, and hospital outcomes in men and women hospitalized with pulmonary embolism (PE) and obstructive sleep apnea in Spain from 2016 to 2018 before and after propensity score matching.

	Before propensity score matching			After propensity score matching		
	Men	Women	p-value	Men	Women	p-value
Age, mean (SD)	66.72 (12.07)	71.38 (11.89)	< 0.001	69.86 (11.31)	71.38 (11.89)	0.005
CCI, mean (SD)	1.15 (1.01)	1.00 (0.88)	< 0.001	1.00 (0.89)	1.00 (0.88)	0.999
AMI, n (%)	67 (4.06)	17 (1.86)	0.003	20 (2.19)	17 (1.86)	0.618
Heart failure, n (%)	213 (12.92)	176 (19.3)	< 0.001	140 (15.35)	176 (19.3)	0.026
PVD n (%)	103 (6.25)	21 (2.3)	< 0.001	19 (2.08)	21 (2.3)	0.749
CVD, n (%)	65 (3.94)	28 (3.07)	0.259	31 (3.4)	28 (3.07)	0.691
Dementia, n (%)	45 (2.73)	25 (2.74)	0.985	30 (3.29)	25 (2.74)	0.494
Rheumatic disease, n (%)	29 (1.76)	21 (2.3)	0.341	19 (2.08)	21 (2.3)	0.749
Liver disease, n (%)	121 (7.34)	48 (5.26)	0.043	53 (5.81)	48 (5.26)	0.609
Diabetes, n (%)	382 (23.17)	260 (28.51)	0.003	241 (26.43)	260 (28.51)	0.319
COPD, n (%)	490 (29.71)	204 (22.37)	< 0.001	215 (23.57)	204 (22.37)	0.540
Renal disease, n (%)	180 (10.92)	100 (10.96)	0.970	91 (9.98)	100 (10.96)	0.491
Cancer, n (%)	226 (13.71)	67 (7.35)	< 0.001	74 (8.11)	67 (7.35)	0.539
Atrial fibrillation, n (%)	185 (11.22)	76 (8.33)	0.021	82 (8.99)	76 (8.33)	0.617
Valvular heart disease, n (%)	93 (5.64)	66 (7.24)	0.109	61 (6.69)	66 (7.24)	0.646
Obesity, n (%)	584 (35.42)	468 (51.32)	< 0.001	422 (46.27)	468 (51.32)	0.031
Coagulopathy, n (%)	33 (2)	15 (1.64)	0.524	15 (1.64)	15 (1.64)	0.999
Pulmonary hypertension, n (%)	164 (9.95)	140 (15.35)	< 0.001	116 (12.72)	140 (15.35)	0.106
Supplemental oxygen, n (%)	113 (6.85)	81 (8.88)	0.063	66 (7.24)	81 (8.88)	0.197
Inferior vena cava filter, n (%)	11 (0.67)	10 (1.1)	0.249	6 (0.66)	10 (1.1)	0.315
Undergone surgery, n (%)	25 (1.52)	10 (1.1)	0.381	16 (1.75)	10 (1.1)	0.236
Thrombolytic therapy, n (%)	92 (5.58)	58 (6.36)	0.421	51 (5.59)	58 (6.36)	0.489
NIV, n (%)	85 (5.15)	67 (7.35)	0.025	53 (5.81)	67 (7.35)	0.186
IV, n (%)	16 (0.97)	10 (1.1)	0.760	4 (0.44)	10 (1.1)	0.107
Non-septic shock, n (%)	9 (0.55)	10 (1.1)	0.120	6 (0.66)	10 (1.1)	0.315
Vasopressors, n (%)	3 (0.18)	2 (0.22)	0.837	0 (0)	2 (0.22)	0.157
LOHS, median (IQR)	7 (6)	8 (6)	0.157	7 (6)	8 (6)	0.160
IHM, n (%)	59 (3.58)	40 (4.39)	0.310	33 (3.62)	40 (4.39)	0.403

*OSA* Obstructive sleep apnea, *CCI* Charlson comorbidity index, *AMI* acute myocardial infarction, *PVD* peripheral vascular disease, *CVD* cerebrovascular disease, *COPD* chronic obstructive pulmonary disease, *NIV* non-invasive ventilation, *IV* invasive ventilation, *LOHS* length of hospital stay, *IHM* in-hospital mortality, *SD* standard deviation, *IQR* Inter quartile range.

p value for comparison of men and women with OSA.

Prior to matching, women were significantly older (71.38 years vs. 66.72 years; p < 0.001) and had a lower mean CCI (1.00 vs. 1.15; p < 0.001). The conditions significantly more frequently coded among women than men were heart failure, diabetes, obesity, and pulmonary hypertension. On the other hand, men had a higher prevalence of acute myocardial infarction, peripheral vascular disease, liver disease, COPD, cancer, and atrial fibrillation. No differences in the use of therapeutic procedures were found between men and women.

Before and after PSM, IHM in women with OSA was similar to that of men with OSA (4.39% vs 3.62%; p = 0.403) (Table 4).

Table 5 shows the results of multivariate logistic regression analysis of variables that were independently associated with IHM for patients with OSA hospitalized with PE and according to sex.

**Table 5:** Multivariable analysis using logistic regression to identify variables associated with in-hospital mortality for patients hospitalized with pulmonary embolism (PE) and obstructive sleep apnea (OSA) according to sex in Spain from year 2016 to year 2018.

Variable	Men	Women	Both sexes
	OR (95% CI)	OR (95% CI)	OR (95% CI)
55–64 years	1.92 (0.39–9.34)	0.87 (0.30–7.42)	1.03 (0.22–4.85)
65–74 years	4.21 (1.06–8.37)	1.23 (0.51–7.15)	3.29 (1.61–6.58)
75–84 years	4.51 (1.91–9.09)	3.7 (1.12–14.59)	3.99 (1.84–9.38)
85 years or over	5.43 (0.97–13.31)	4.39 (0.73–16.34)	4.9 (1.25–8.96)
Heart failure	NS	NS	1.79 (1.54–2.47)
Dementia	NS	4.13 (1.26–13.52)	3.97 (1.65–9.53)
COPD	2.84 (1.32–5.78)	NS	NS
Cancer	5.45 (3.06–9.73)	3.08 (1.01–7.88)	3.94 (1.89–5.43)
Atrial fibrillation	3.57 (1.92–6.64)	3.92 (1.74–8.85)	4.91 (2.76–8.73)
IV	9.61 (1.96–27.87)	16.31 (3.7–41.83)	13.33 (4.97–30.36)
Non-septic shock	8.03 (1.21–33.21)	11.12 (2.11–38.44)	9.88 (2.47–29.51)
Women	NA	NA	1.18 (0.72–1.96)

*COPD* Chronic obstructive pulmonary disease, *IV* invasive ventilation, *OR* Odds ratio, *CI* confidence interval, *NA* not applicable, *NS* not significant.

The risk of dying in hospital rose with age for both sexes. Cancer, atrial fibrillation, non-septic shock, and mechanical ventilation increased IHM in both men and women with OSA hospitalized with PE.

Women with OSA who had heart failure or dementia coded in their discharge report had a higher risk of dying in hospital. For men, COPD was associated with higher IHM.

As found in the PSM, the logistic regression model showed no sex differences between patients with OSA.

## Discussion

In this nationwide population-based study, OSA affected 5.47% of PE patients. Consistent with the general population, the prevalence of OSA was about twice as high in men as in women^16^, indicating that the effect of OSA on the risk of PE is not affected by sex. On the other hand, no association was detected between OSA and higher IHM in men or in women admitted for PE.

We found that the prevalence of OSA in patients with PE was consistent with that described by Le Mao et al.^15^, who reported this disease to be relatively infrequent in people with PE. Other studies have reported figures ranging from 15 to 65%, although the results may be confounded as a result of selection and detection biases^6,17,18^.

The mechanisms underlying the association between OSA and PE remain unclear. It has been speculated that OSA leads to the development of PE through the three mechanisms of Virchow’s classic triad, namely, vascular endothelial injury, stagnant blood flow, and hypercoagulable blood status^8^. OSA and PE share a series of risk factors, including obesity and increasing age. Furthermore, the sedentary lifestyle associated with OSA and PE can lead to venous stasis and thrombosis^19^. Recurrence of hypoxia and reoxygenation in OSA can increase reactive oxygen species and inflammatory mediators and therefore reduce the availability of nitric oxide and impair vascular endothelial function^8^. Moreover, fibrinogen levels and platelet activity are increased, whereas fibrinolytic capacity is lower in patients with OSA than in healthy controls. The abovementioned factors can contribute to the hypercoagulable state of patients with OSA, which may in turn mediate the need for higher doses of warfarin in patients with PE^20^.

Patients with PE complicated by OSA have more severe conditions than their non-OSA counterparts^21,22^, possibly owing to nocturnal hypoxemia or OSA-related hypercoagulability^23^. However, a recent systematic review and meta-analysis revealed no significant differences between PE patients with and without OSA in the right ventricle to left ventricle ratio or in the length of stay^24^. Similarly, Le Mao et al.^15^ did not find significant differences in the rates of echocardiographic right ventricle dysfunction between both groups. PE-related right ventricle strain might be considerably better offset in patients with pronounced OSA, as they are adapted to repetitive right heart pressure overloads caused by impaired pulmonary perfusion resulting from repetitive oxygen desaturation during sleep^21,25^. Consequently, their right ventricles may be less prone to injury and hemodynamic collapse as a consequence of acute PE. The potential resulting protective effect could be associated with lower mortality. This explanation could justify the findings of Joshi et al.^26^, who reported prevalent OSA to be associated with lower IHM in PE patients. The same authors also suggested that OSA could lead to increased hemoglobin levels resulting from hypoxemia, which may confer a protective effect in acute PE by preventing hypoxia from worsening^14,26^.

Data from regular and critical care wards show a decreased mortality risk in hospitalized patients with OSA and associated diseases, such as pneumonia^27,28^, cardiovascular disease^29^, ischemic stroke^30^, and subarachnoid hemorrhage^31^. These results point to a potential protective effect of OSA with respect to mortality, probably as a result of ischemic preconditioning or a higher level of care or vigilance in these patients^32^. It is also important to bear in mind the obesity paradox, by which obese patients fare better in the face of acute illness, possibly because they seek medical care earlier or have increased metabolic reserves^27^. However, while obesity was more frequent in men and women with OSA than in those without the disease, the difference was not maintained after PSM. Ghiasi et al.^14^ also analyzed the relationship between OSA and 30-day mortality in PE patients and found that OSA did not affect mortality directly. In fact, it was affected by complications of OSA such as hypertension and thrombosis. In addition, our study demonstrated that OSA was not associated with higher IHM in men or in women admitted for PE.

As might be expected, NIV was more common in men and women with OSA than in those without OSA, possibly because the former may have had more severe PE or hypoxemia^26^. Nevertheless, NIV was not a predictor of IHM in OSA patients admitted with PE.

The variables associated with IHM for men and women hospitalized for PE and OSA in our study were older age, cancer, atrial fibrillation, non-septic shock, and invasive mechanical ventilation. Among women with OSA, heart failure was also associated with a higher risk of dying in hospital. Similarly, Roca et al.^33^ demonstrated that OSA is associated with incident heart failure or death in women but not in men. For men, we also found that COPD was associated with higher IHM, as previously reported globally after PE^34^. In this sense, Xie et al.^35^ found that the association between concomitant OSA and COPD and PE (overlap syndrome) was significant only in the male subgroup, in contrast with the female group. Moreover, the authors observed that patients with overlap syndrome had a lower degree of oxygen saturation during sleep and a higher probability of PE than controls and patients with OSA alone.

Our study is subject to a series of limitations. First, given the nature of our database, OSA was not confirmed by polysomnography, which is the gold standard for diagnosis. As a consequence, we do not have information on the severity of OSA, which has demonstrated prognostic value in PE^36^. Second, the absence of laboratory and imaging data and data on medical treatment during hospital stay prevents us from predicting outcomes in PE^1^. Third, we had no data on use of continuous positive airway pressure (CPAP), which is the primary treatment for OSA and may reduce exaggerated coagulant activity and platelet function and improve fibrinolytic capacity in affected patients^19^. Finally, mortality data are valid only for inpatient mortality and all-cause mortality. Despite these limitations, our data on the relationship between OSA and PE are highly reliable because of the validity of the Spanish National Hospital Discharge Database (SNHDD) and the large sample size evaluated.

In conclusion, our study showed that the effect of OSA on the risk of PE was not affected by sex and that the presence of OSA was not associated with higher IHM in men or in women admitted for PE. The variables associated with IHM for men and women hospitalized for PE and OSA in our analysis were older age, cancer, atrial fibrillation, non-septic shock, and invasive mechanical ventilation. Recognizing an association between these two diseases is important and could improve prognosis, since CPAP may decrease the incidence or recurrence of PE in OSA patients.

## Methods

### Design, setting, and participants

We conducted a retrospective epidemiological study. Participant information was obtained from the SNHDD.

Spanish legislation requires all public and private hospitals to provide the Ministry of Health with information regarding all discharges. Patient information includes age, sex, province of residence, and admission and discharge dates, up to 20 diagnoses, and a maximum of 20 diagnostic or therapeutic procedures conducted during admission. Information regarding the discharge destination was also collected. The 10th Revision of the International Classification of Diseases (ICD-10) has been used by the SNHDD since 2016 to code diagnoses and procedures. More details regarding the SNHDD can be found elsewhere^37^.

### Study population

The study population comprised patients aged ≥ 18 years who were discharged from a Spanish hospital with a primary diagnosis of PE (ICD 10 codes I26.92 and I26.99) between January 1, 2016 and December 31, 2018. The primary diagnosis was the clinical condition requiring the patient to be admitted to hospital^37^.

Patients with codes for septic or iatrogenic PE, acute cor pulmonale, or PE secondary to obstetric complications (ICD10 codes are shown in Supplementary Table 1) were excluded following the recommendations of Smith et al.^38^.

The study population was stratified according to the presence of OSA. The ICD10 code used to identify patients with OSA in any diagnosis field (positions 2–20) was G47.33.

### Study variables

The main study outcome measures were the prevalence of OSA in patients hospitalized with PE, use of therapeutic procedures, LOHS, and IHM.

Age was categorized into 5 groups (18–54 years, 55–64 years, 65–74 years, 75–84 years, and ≥ 85 years).

The comorbidities included in the CCI were extracted using the algorithms proposed by Quan et al.^39^ for administrative databases coded based on ICD 10.

The conditions included in the CCI were analyzed independently and categorized according to the number (CCI = 0, CCI 1–2, and CCI > 2).

The CCI conditions shown in Supplementary Table 1 are complemented by the codes used to identify various diagnoses (atrial fibrillation, valvular heart disease, obesity, coagulopathy, pulmonary hypertension, and non-septic shock) and therapeutic procedures (dependence on supplemental oxygen, inferior vena cava filter, thrombolytic therapy, NIV, invasive ventilation, and use of vasopressors), which were also analyzed.

The variable “Undergone surgery” included those patients who had undergone a surgical procedure during their hospital stay with PE.

### Propensity score matching

As can be seen in Table 1, the characteristics of the OSA and non-OSA populations differ significantly with respect to age, sex, and CCI. We used PSM to make baseline characteristics more similar, by matching each man and woman with OSA with a non-OSA man and woman, respectively. The propensity score used for matching was obtained from a multivariable logistic regression model that included age and all comorbid conditions present at admission. To analyze sex differences in the co-occurrence of PE with OSA, we also matched men with women affected by both conditions. These methods have been reported elsewhere^40,41^.

### Statistical methods

We show absolute frequencies and proportions for categorical variables and means with standard deviations (SD) or medians with interquartile ranges (IQR) for continuous variables.

Patient demographic characteristics, comorbidities, procedures, and hospital outcomes were compared between patients with and without OSA according to sex. The *t* test or Wilcoxon rank-sum test was used for continuous variables and the chi-squared or Fisher exact test for categorical variables.

We constructed multivariable logistic regression models to identify those variables that were independently associated with IHM in patients with PE and OSA according to sex. The results are presented as odds ratio (OR) with the 95% CI.

Stata version 14 (Stata, College Station, Texas, USA) was used for PSM and all data analyses.

### Ethical aspects

The SNHDD is provided free of charge by the Spanish Ministry of Health to any investigator who sends a justified request^42^. As this database is mandatory and anonymized, according to the Spanish legislation, it is not necessary to obtain ethics committee approval.

## Supplementary Information

Supplementary Table 1.

## Data Availability

"No additional data available". According to the contract signed with the Spanish Ministry of Health and Social Services, which provided access to the databases from the Spanish National Hospital Discharge Database (SNHDD), we cannot share the databases with other investigators and must delete the databases once the investigation has concluded. Consequently, we cannot upload the databases to a public repository. However, any investigator can apply for access to the databases by filling out the questionnaire available at: http://www.msssi.gob.es/estadEstudios/estadisticas/estadisticas/estMinisterio/SolicitudCMBDdocs/Formulario_Peticion_Datos_CMBD.pdf. All other relevant data are included in the paper.
