# Supplementary material for: Sex-differences in the effect of obstructive sleep apnea on patients hospitalized with pulmonary embolism and on in-hospital mortality
Source: Sci Rep. 2021 Sep 15;11:18390. doi: 10.1038/s41598-021-97923-y (PMC8443545; doi:10.1038/s41598-021-97923-y)
Supplement: Supplementary file 1 — Supplementary Table 1. [file 41598_2021_97923_MOESM1_ESM.docx]

**Supplementary Table 1:** ICD-10 codes for the clinical diagnosis and procedures used in this investigation.

|  | ICD-10 |
| --- | --- |
| Acute cor pulmonale* | I26.09 |
| Iatrogenic PE and infarction* | I26.90; I26.99 |
| Septic PE* | I26.90 |
| PE Complicating abortion* | O03.7, O03.2, O03.7, O08.2, O88.019, O88.011, O88.012, O88.013, O88.02, O88.03 |
| Atrial fibrillation | I48.0, I48.1, I48.2, I48.91 |
| Valvular heart disease | I05.X, I06.X, I07.X, I08.X, I34.X,I35.X, I36.X, I37.X |
| Obesity | E66.X |
| Coagulopathy | D68.XX |
| Pulmonary hypertension | I27.2 |
| Dependence on supplemental oxygen | Z99.81 |
| Inferior vena cava filter placement | 02HV0DZ, 02HV3DZ, 02HV4DZ, 02LV0CZ, 02LV0DZ, 02LV0ZZ, 02LV3CZ, 02LV3DZ, 02LV3ZZ, 02LV4CZ,,02LV4DZ,,02LV4ZZ,,02VV0CZ , 02VV0DZ, 02VV0ZZ, 02VV3CZ, 02VV3DZ , 02VV3ZZ, 02VV4CZ, 02VV4DZ , 02VV4ZZ , 6H00DZ, 06H03DZ , 06H04DZ , 06L00CZ, 06L00DZ , 06L00ZZ , 06L03CZ, 06L03DZ , 06L03ZZ , 06L04CZ, 06L04DZ , 06L04ZZ , 06V00CZ, 06V00DZ ,06V00ZZ 06V03CZ, 06V03DZ, 06V03ZZ, 06V04CZ, 06V04DZ, 06V04ZZ |
| Thrombolytic therapy | 3E03317, 3E04317, 3E05317, 3E06317, 3E08317 |
| Non-invasive mechanical ventilation | 5A09357, 5A09457, 5A09557 |
| Non-septic shock | R57.0, R57.1, R57.8, R57.9, |
| Vasopressors medication | 3E030XZ, 3E033XZ, 3E040XZ, 3E043XZ, 3E050XZ, 3E053XZ, 3E060XZ, 3E063XZ |

PE: Pulmonary embolism.

*ICD-10 codes used to define pulmonary embolism for study purpose based on Smith et al .(Smith SB, Geske JB, Kathuria P, Cuttica M, Schimmel DR, Courtney DM, Waterer GW, Wunderink RG. Analysis of national trends in admissions for pulmonary embolism. Chest. 2016;150(1):35-45. )
